# Sequencing of Chloroplast Genome Using Whole Cellular DNA and Solexa Sequencing Technology

**DOI:** 10.3389/fpls.2012.00243

**Published:** 2012-11-08

**Authors:** Jian Wu, Bo Liu, Feng Cheng, Nirala Ramchiary, Su Ryun Choi, Yong Pyo Lim, Xiao-Wu Wang

**Affiliations:** ^1^Institute of Vegetables and Flowers, Chinese Academy of Agricultural SciencesBeijing, China; ^2^Department of Horticulture, Plant Genome Research Institute, Chungnam National UniversityDaejeon, South Korea

**Keywords:** chloroplast genome, sequencing, Solexa sequencing technology, whole cellular DNA, *Brassica rapa*

## Abstract

Sequencing of the chloroplast (cp) genome using traditional sequencing methods has been difficult because of its size (>120 kb) and the complicated procedures required to prepare templates. To explore the feasibility of sequencing the cp genome using DNA extracted from whole cells and Solexa sequencing technology, we sequenced whole cellular DNA isolated from leaves of three *Brassica* *rapa* accessions with one lane per accession. In total, 246, 362, and 361 Mb sequence data were generated for the three accessions Chiifu-401-42, Z16, and FT, respectively. Micro-reads were assembled by reference-guided assembly using the cpDNA sequences of *B. rapa*, *Arabidopsis thaliana*, and *Nicotiana tabacum*. We achieved coverage of more than 99.96% of the cp genome in the three tested accessions using the *B. rapa* sequence as the reference. When *A. thaliana* or *N. tabacum* sequences were used as references, 99.7–99.8 or 95.5–99.7% of the *B. rapa* cp genome was covered, respectively. These results demonstrated that sequencing of whole cellular DNA isolated from young leaves using the Illumina Genome Analyzer is an efficient method for high-throughput sequencing of cp genome.

## Background

The chloroplast (cp) genome contains a wealth of information that has been shaped by speciation, rendering it a rich resource to trace population-level processes and evolutionary divergence. Therefore, the cp genome sequence is very important in several fields of plant biology, including phylogenetics, molecular biology, evolutionary biology, and cp genetic engineering. Complete sequences of cp genomes were first reported for tobacco and a liverwort in 1986 (Ohyama et al., 1986; Shinozaki et al., 1986). Since then, the sequences of cp genomes from a number of land plants and algae have been determined. However, there are still challenges in rapid and cost-effective sequencing or re-sequencing of the cp genome.

Traditional sequencing begins with the construction of plastid or other genomic libraries. Construction of these resources is a difficult part of this work, since it is complicated to isolate cps and construct libraries. For taxa that are rare and/or difficult to obtain, large-scale isolation of cps would be one difficulty in the conventional sequencing methodology. Therefore, a method to sequence cp genomes with a simple and rapid sample preparation step would greatly benefit research that requires cpDNA sequencing.

Because of the characteristics of conserved genome size, gene arrangement, and coding sequences among cp genomes, a PCR-based approach has been used for their amplification, sequencing, and assembly (Dhingra and Folta, 2005; Cronn et al., 2008). This method is useful for obtaining cpDNA sequences from divergent species. However, the complex PCR procedure and the large number of PCR reactions required to cover the cp genome of >120 kb limits the application of this approach. Therefore, there is a need for a simpler method to sequence cp genomes. Since whole cellular DNA contains chromosomal DNA, mitochondrial DNA, and cp DNA, any of the three types of DNA sequence could be derived from sequencing this DNA material. The fact that the grapevine cp genome sequence was obtained as a byproduct of whole genome sequencing indicates that it is possible to use total DNA for cp genome sequencing (Velasco et al., 2007). To ensure accurate assembly, a sufficient sequencing depth is required. However, this is difficult to achieve using traditional Sanger sequencing technology.

The development of next-generation sequencing technologies has shed new light on easy sequencing of complete cp genomes. Moore et al. (2006) were the first to attempt to use second generation sequencing technology (a 454 GS 20 system) for the cp genome. Cronn et al. (2008) developed a multiplex sequencing-by-synthesis approach to sequence cp genomes. That method combined PCR-amplified cp genomes and indexing tags for Solexa sequencing. Both of these studies demonstrated that second generation sequencing technology is a powerful tool for sequencing plastid genomes quickly and economically. However, both methods required substantial work to prepare sequencing templates either for traditional plasmid isolation or to obtain PCR-amplified cpDNA fragments.

In this study, using routinely isolated whole cellular DNA from young leaves of *Brassica rapa*, we generated highly accurate and essentially complete cp genome sequences by Illumina GA II. Our results show that this method is faster and more economical than traditional methods, and can be used for rapid sequencing of cp genomes.

## Materials and Methods

### DNA sources

Three *B. rapa* accessions, Chiifu-402-41, Z16, and FT were used for sequencing of the cp genome. Chiifu-402-41, the material used for whole genome sequencing (Wang et al., 2011), was donated by the Korea *Brassica* Genome Resource Bank. The accession Z16 is a Chinese cabbage line (ssp. *pekinensis*) obtained from the Chinese Academy of Agricultural Sciences (CAAS). The accession FT (CGN1010) is a fodder turnip (ssp. *rapa*) obtained from the Dutch Crop Genetic Resources Center (CGN), Wageningen, The Netherlands. DNA was isolated from young leaves using a modified CTAB method (Fulton et al., 1995).

### DNA sequencing

The isolated whole cellular DNA was sheared, polished, and prepared according to Illumina Sample Preparation kit (Solexa Inc, 2007). The nucleotide sequence was determined according to the Solexa sequencing method (Wang et al., 2008). Sequencing was carried out with one accession per lane to generate 35-mer micro-reads.

### Reference-guided assembly for cp genome sequence

**Figure 1:**
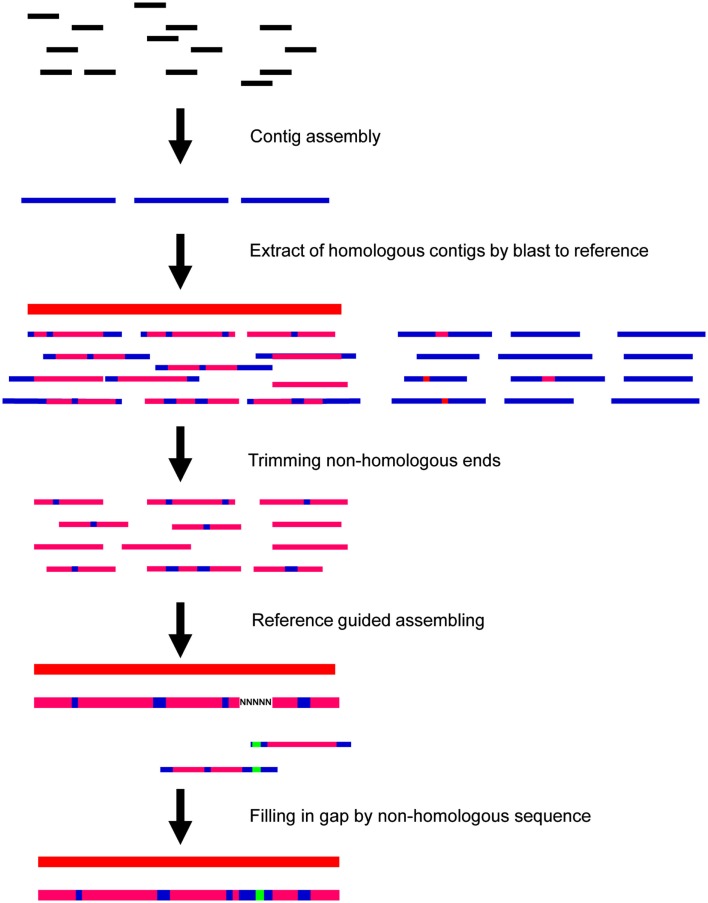
**Schematic view of micro-reads assembly method for chloroplast (cp) genome**. *De novo* assembled contigs (blue bars) are aligned to reference (red bar) to extract sequences generated from cp genome (thin pink bars). Draft consensus (thick pink bar) was constructed guided by reference. Gaps were filled by extending sequence and joining two contigs that overlapped (green bar) by 10 or more nt.

After the run, fluorescent images were processed into sequences using the Illumina/Solexa Pipeline (version 0.2.2.6). Reads containing “N” were filtered out before further analyses. The strategy used for cp genome assembly is outlined in Figure 1. The micro-reads (35-mer) were first *de novo* assembled using SOAPdenovo (Li et al., 2010) with an overlap *k*-mers value of 27. Second, contigs longer than 50 nt were extracted to construct the consensus using our reference-guided assembly (RGA) program. During the RGA process, all contigs were aligned to the reference cp genome sequence by BLASTN (http://www.ncbi.nlm.nih.gov). Contigs with matches greater than 80% were selected for assembly after trimming non-homologous ends. The trimmed fragments of 50 nt or longer were assembled under the guidance of the reference. If there was no overlap between two adjoining fragments, the interval was filled with “N” to match the length in the draft consensus. To fill these gaps, the corresponding two contigs of each gap were extended to the end and merged if there was an overlap of 10 or more bp (Figure 1).

To explore the applicability of this cp genome sequencing strategy, we compared the assemblies guided by cp genome sequences of *B. rapa* (GenBank: DQ231548), *Arabidopsis thaliana* (GenBank: NC000932; Sato et al., 1999), or *Nicotiana tabacum* (GenBank: NC001879; Shinozaki et al., 1986).

### Gene annotation

The *B. rapa* cp genes were annotated using DOGMA (Wyman et al., 2004). This program used a FASTA formatted input file of the complete cp genome sequences and identified putative protein-coding genes by performing BLASTX searches against a custom database of previously published cp genomes. Both transfer RNA and ribosomal RNA were identified by search against *A. thaliana* genes database using BLASTN.

### Sequence validation

To assess the accuracy of this approach for sequencing cp genome, the consensus assembled for Chiifu-402-41 cp was mapped against the sequence derived from the Sanger-based sequencing method (GenBank: DQ231548). We used BLASTN (http://www.ncbi.nlm.nih.gov) to identify mismatches between the two sequences. Once a mismatch was identified, we designed sequence-specific primers to amplify the inconsistent region and sequenced the fragment using the traditional Sanger sequencing method.

### Polymorphism discovery in the *B. rapa* cp genome

To assess the applicability of this approach for re-sequencing of the cp genome, we used all the *de novo* assembled contigs to align onto the reference cp genome sequence (GenBank: DQ231548) to identify single nucleotide polymorphisms (SNPs) and insertion/deletion polymorphisms (InDels) among the three accessions.

To identify the SNPs based on short reads aligning, we used short reads of Z16 and FT accessions to respectively align to reference sequence of Chiifu-402-41 cp genome based on SOAP with default parameter (Li et al., 2008). And then, using these results of reads aligning, we identified SNPs between Chiifu-402-41 vs. Z16 and Chiifu-402-41 vs. FT by SOAPsnp with default parameter (Li et al., 2009).

## Results

### Analysis of micro-reads generated from whole cellular DNA

**Figure 2:**
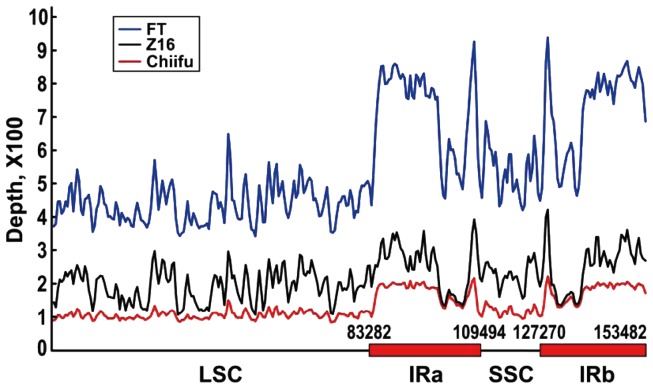
**Plot showing sequencing depth by position for chloroplast genomes of three *B. rapa* accessions sequenced by Solexa Genome Analyzer with whole cellular DNA as template**. Number of micro-reads per position (*y*-axis) is plotted against position in the assembly (*x*-axis, in kb) in a window size of 100 bp. Numbers above *x*-axis indicate boundary sites of large single copy (LSC) and small single copy (SSC), and two inverted repeats (IRa and IRb).

One lane Solexa sequencing of whole cellular DNA produced 7,015,639, 10,313,714, and 10,356,209 35-mer micro-reads for Chiifu-402-41, Z16, and FT, respectively, corresponding to the total sequenced length of 246, 362, and 361 Mb, respectively (Table 1). Of the total reads, 9.3% (653,057), 26.4% (2,721,148), and 10.4% (1,073,449) were mapped to the reference cp genomes for Chiifu-402-41, Z16, and FT, respectively, corresponding to an overall average sequencing depth of 103×, 550×, and 217×, of their cp genome, respectively (Figure 2). cp Genomes of most land plants have a quadripartite structure with large and small single copy regions (LSC and SSC) separated by two copies of large inverted repeats (IRa and IRb). The *B. rapa* cp genome is 153,482-bp long; the LSC is 83,282 bp, the SSC 17,776 bp, and each of the two IR copies is 26,212 bp (Figure 2). The depth of the reads showed two peaks in the corresponding inverted repeat regions after being aligned to the reference genome. This was consistent with the structure of the cp genome.

**Table 1:** **Characteristics of reads from one lane sequencing on Illumina Solexa 1G Genome sequencer**.

	Chiifu-402-41	Z16	FT
Total reads	7,015,639	10,313,714	10,356,209
Aligned reads	653,057	2,721,148	1,073,449
Aligned ratio (%)	9.3	26.4	10.4
Mean read depth (-fold)	103	550	217
N50 (bp)	13,509	3997	7461

*Mean read depth was calculated by including one copy of inverted repeats*.

### Contig assembly using a reference-guided approach

Small reads were first assembled into contigs, from which very short N50 lengths (length of the shortest contig among those that collectively covered 50% of the assembly) were derived for all three accessions. For the “best assembly” lane of the Chiifu-402-41 accession, there were 25,493 contigs with an N50 of 82 nt. In the second assembly step, the number of contigs that met the conditions of longer than 50 bp and greater than 80% identity to the *B. rapa* reference cp genome sequence decreased to 22 with an N50 of 13,509 nt. Z16 had 86 contigs and an N50 of 3,997 nt; FT had 46 contigs and an N50 of 7,461 nt (Table 1). The contigs were trimmed from the positions of the first or the last hit nucleotide to build a draft consensus. Finally, to fill gaps in the consensus, the sequence of the two corresponding contigs in the direction of the gap were compared. If there was an overlap of 10 bp or more, the two contigs were joined together. Using this strategy, we achieved a minimum coverage of 99.96% of the cp genome for the *B. rapa* accessions Chiifu-402-41, Z16, and FT, with one, seven, and five gaps, respectively (see Table S1 in Supplementary Material for positions and lengths of gaps). The only gap in the consensus of Chiifu-402-41 was also present in the other two accessions. When went through the sequence of region where the gap located, it revealed that there was a satellite sequence of (tggatatagactcatgaaag)_3_. The common 8-bp gap was located in the second repeat of the satellite.

**Figure 3:**
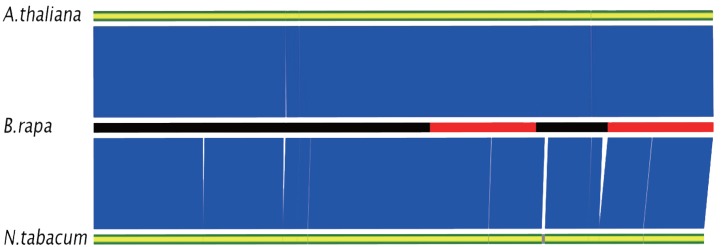
**Comparison of assemblies for Chiifu-402-41 guided by chloroplast genome sequences of *B. rapa* (DQ231548), *A. thaliana* (NC000932), and *N. tabacum* (NC001879)**. Assembled consensuses are represented by three bars. For *B. rapa*, black parts of bar indicate LSC or SSC; red parts of bar indicate IRa or IRb. For *A. thaliana* and *N. tabacum*, silver parts indicate gaps. Blue block between bars of *B. rapa* and *A. thaliana* or *N. tabacum* indicates identity >95%.

To evaluate the applicability of this approach to sequencing those species without known cp sequences, we assembled consensuses using *A. thaliana* or *N. tabacum* as a reference and then compared the completeness of these assemblies. Guided by the cp genome sequence of *A. thaliana* and *N. tabacum*, the assembled contigs covered 99.83 and 97.77% of the cp genome from *B. rapa* accession Chiifu-402-41, respectively (Table 2; Figure 3). Among the three sequenced accessions, the lowest coverage in FT was 99.73%, which was obtained using the *A. thaliana* sequence as the reference. The lowest coverage in Z16 was 95.55%, which was obtained using the *N. tabacum* sequence as the reference. In assemblies guided by the *A. thaliana* sequence, there were four, seven, or six gaps in the consensus of Chiifu-402-41, Z16, or FT, respectively. In assemblies guided by the *N. tabacum* cpDNA sequence, there were 4, 21, or 10 gaps in the consensus of Chiifu-402-41, Z16, or FT, respectively. Table S1 in Supplementary Material shows the positions and lengths of the gaps. None of the gaps was common between the assemblies guided by *A. thaliana* or *N. tabacum*.

**Table 2:** **Assembly of chloroplast genomes of three *B. rapa* accessions guided by cp genome sequence of *B. rapa* (DQ231548), *A. thaliana* (NC000932), and *N. tabacum* (NC001879)**.

Accession	*B. rapa*			*A. thaliana*				*N. tabacum*			
	Coverage (%)	No. of gaps	Total length of gaps (bp)	Coverage (%)	No. of gaps	Total length of gaps (bp)	Error base	Coverage (%)	No. of gaps	Total gap length (bp)	Error base
Chiifu	99.99	1	8	99.83	4	156	0	97.77	4	1048	0
Z16	99.96	7	52	99.74	7	310	12	95.52	21	4161	34
FT	99.96	5	51	99.73	6	481	9	96.19	10	2339	3

**Figure 4:**
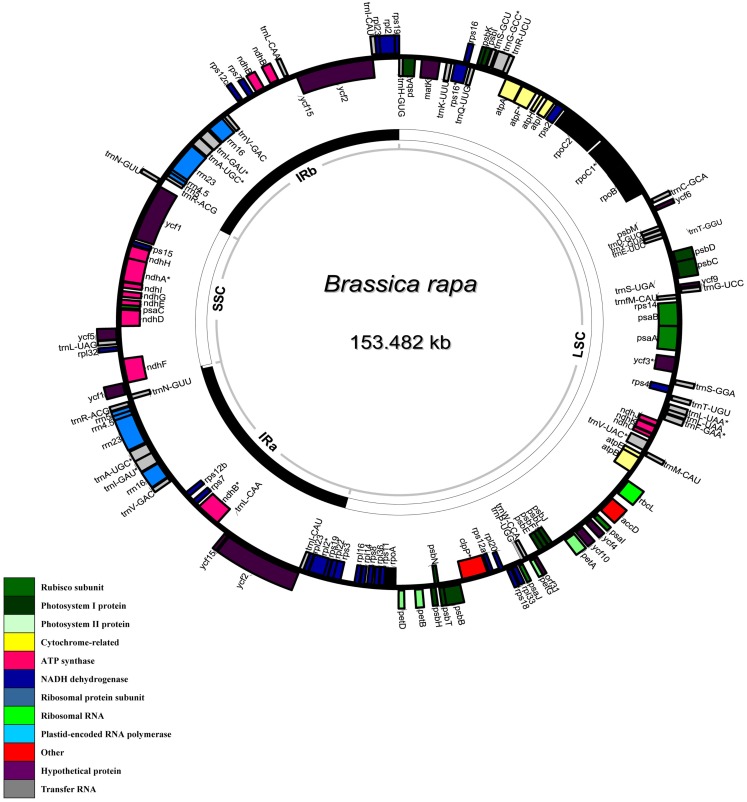
**Circular gene map of *Brassica rapa* chloroplast genome**. The thick lines indicate the extent of the inverted repeats (IRa and IRb, 26,213 bp), which separate the genome into small (SSC, 17,777 bp) and large (LSC, 83,282 bp) single copy regions. Genes on the outside of the map are transcribed in the clockwise direction and genes on the inside of the map are transcribed in the counterclockwise direction.

Using DOGMA (Wyman et al., 2004) software, a total of 89 potential protein-coding genes (including eight genes duplicated in the inverted repeat), 8 ribosomal RNA genes and 37 transfer RNA genes were assigned to the genome on the basis of similarities to the cp gene previously reported in other species (Figure 4; Table S2 in Supplementary Material). Fourteen genes contained one intron, while one gene had two introns. In addition, the number of genes in *B. rapa* cp genome was similar to *A. thaliana* (a total of 128 genes including 89 protein-coding genes, 4 rRNA genes, and 37 tRNA genes; Sato et al., 1999), while it was distinctly more than that in the *N. tabacum* cp genome (84 genes were identified in total; Shinozaki et al., 1986).

No base error was observed in the assemblies guided by either *A*. *thaliana* or *N*. *tabacum* for Chiifu-402-41. However, several base errors were observed in the assemblies of FT and Z16 (Table 2; see Table S2 in Supplementary Material for positions of errors). The rate of base error was 0.0078% for Z16 and 0.0058% for FT when guided by *A. thaliana*, and 0.022% for Z16 and 0.0019% for FT when guided by *N. tabacum*. The errors located at 37 bp and 57 bp were appeared in both assemblies of FT and Z16 guided by either *A*. *thaliana* or *N. tabacum*.

A fragmented loss of sequence from the consensus was observed for all three *B. rapa* accessions when using *A. thaliana* or *N. tabacum* as the reference (see Table S3 in Supplementary Material for the positions of InDel). To analyze this further, we aligned the cp sequence of *B. rapa* with those of *A. thaliana* and *N. tabacum*. This revealed that this type of error was resulted from sequence deletions in cp genomes of *A. thaliana* and *N. tabacum*. Since this type of error was reflected by calculating coverage, it was not included in the number of base errors.

### Sequence validation

Comparison of the consensus of Chiifu-402-41 assembled in this study with the reference sequence generated earlier by Sanger sequencing revealed one base difference (Ref: G/Solexa: A) at position of 116,457 bp at a sequencing depth of 72× by Solexa. To confirm the nucleotide at this position, we designed primers to amplify the inconsistent region. We amplified a 508-bp cp DNA fragment using primers 5′-AAAATCATTCGTGGTAA-3′ (forward) and 5′-AAATCATTGCTTCATCTA-3′ (reverse). The produced fragment was later sequenced by conventional Sanger sequencing. The result showed that the nucleotide at the mismatched position should be A, and not G, in the reference cp sequence of Chiifu-402-41.

### Polymorphisms identified in re-sequencing

To identify the sequence polymorphisms of cp genomes within *B. rapa* species, we mapped all *de novo* assembled contigs against the reference cp sequence (DQ231548). The hit contigs covered 100% of the cp genome for all three accessions. Comparison of Z16 and FT cp genome sequences with that of the reference cp sequence of Chiifu-402-41 revealed 31 and 8 SNPs, respectively (Table 3). Only 1-bp InDels were identified among the three accessions. Four InDels were observed either between Chiifu-402-41 and Z16 or between Chiifu-402-41 and FT when their consensus cpDNA sequences were compared (Table 3; see Table S4 in Supplementary Material for InDel positions). Amongst these SNPs and InDels, there were 14, 19, 3, and 7 mutations located in LSC, IRa, SSC, and IRb, respectively. Particularly, 27 of these 43 (63%) mutations located in nine genes of *B. rapa* cp genome (*trnH*-GUG, *rpoC2*, *rpoC1*, *rps19.1*, *rpl2.1*, *rrn23*, *psaC*, *ycf1.2*, and *rrn23*; see Table S4 in Supplementary Material).

**Table 3:** **Sequence polymorphisms identified by reference-guided assembly of cp genomes of *B. rapa***.

	Chiifu-402-41	Z16	FT
No. of SNP	1	31	8
No. of InDel	0	4	4

*No. of SNPs (single nucleotide polymorphisms) and No. of insertions/deletions (InDels) are those compared with reference (DQ231548)*.

We further compared the efficiency in sequence variants discovery by alignment of *de novo* assembled consensus with alignment of short reads. Using SOAPsnp (Li et al., 2009), a total of 10 SNPs were identified between Chiifu-402-41 and Z16, and 5 SNPs were identified between Chiifu-402-41 and FT. Comparing to SNPs identified by consensus aligning method, seven SNPs between Chiifu-402-41 and Z16, and two SNPs between Chiifu-402-41 and FT were same in genotype in both methods (see Table S4 in Supplementary Material). To confirm the nucleotides at the positions where different nucleotides were indicated by two methods, we randomly selected two positions (at the positions of 66,083–66,086 and 131,065) to design primers to amplify the inconsistent regions in Z16 accession (see Table S4 in Supplementary Material for primer sequences). The result from traditional Sanger sequencing showed that at the position 66,083–66,086 the 4 nt were “TTCT” which were consistent to those by consensus aligning method, however, the nucleotide at the position of 131,065 was “C” which was same as the result from reads aligning method.

## Discussion

In this study, we demonstrated the feasibility of sequencing the cp genome using whole cellular DNA and reference-guided *de novo* assembly of micro-reads into complete cp genome sequences. In light of the challenges associated with established methods for cp genome sequencing, micro-read sequencing of whole cellular DNA is a rapid and cost-effective approach for sequencing/re-sequencing of cp genomes.

Large-scale isolation of chloroplasts is one of the laborious works in traditional cp genome sequencing methods. To avoid this problem, we used whole cellular DNA instead of cpDNA as the sequencing template. Unlike the alternative PCR-based methods reported previously (Dhingra and Folta, 2005; Cronn et al., 2008), this method also avoids the possibility of introducing base errors during PCR reactions.

The cp genome is well-conserved in terms of size, gene arrangement, and coding sequences, at least within major subgroups of the plant kingdom. This is the basis for this whole cellular DNA sequencing strategy, as it allows micro-reads to be assembled correctly using a reference-guided method. As expected, the closer the phylogenetic relationship between the reference and the target, the better the coverage of the assembled cp genome. However, our results of greater than 99% coverage when the assembly was guided by the *A. thaliana* cp DNA sequence and greater than 95% coverage when guided by the *N. tabacum* cp genome sequence indicated that this method could be also used for species without a closely related species in the plastid genome database. More than 150 cp genome sequences from plants belonging to 124 genera have been deposited in GenBank. This provides a base for widespread use of this approach to assemble draft cp genome sequences.

For the cp genome assembly, instead of the strategy of *de novo* assembling contigs which we used in the present study, small reads alignment to reference genome is an alternative approach. One can expect that the read mapping approach is computationally less demanding and faster than *de novo* assembling. Moreover, it has the advantage that the read coverage information can be used for reliable detection of sequence variation. However, when there are InDels or structural variations in cp genomes, which have been reported for nine grass species (Golenberg et al., 1993), Korean ginseng (Kim and Lee, 2004), *Pinus* (Parks et al., 2009), the strategy of assembling contigs is very important to identify exact mutations between cp genomes. Additionally, for the species without known cp genome sequence, aligning short reads to cp genome of other plant species was impractical based on short reads aligner, especially for the high ratio polymorphism regions, because the reads from repeated regions or homologous regions cannot be distinguished. Furthermore, conventional Sanger sequencing result on two positions (66,083 and 131,065 bp) indicated that both consensus aligning method and reads aligning method existed false positive in SNP identification. To improve the veracity in sequence variant identification, generating pair-ends reads is a potential approach.

Although we obtained reasonably high coverage of the cp genome using the present approach, there were a number of gaps in the consensus sequence. Taking the assembly of Chiifu-402-41 as an example, when the *B. rapa* cp genome sequence was used as the reference, there was an 8-bp gap in a region of three 20-bp repeats, and this gap was also present in the other two accessions. This is caused by the limitations of the assembly software itself because of the *k*-mer value used in the assembly, rather than overlapping of micro-reads. However, when the contigs shorter than 50 bp were aligned to the reference, this gap was filled by a 42-nt contig with overlaps to the two adjoining contigs. In the assemblies guided by sequences of *A. thaliana* or *N. tabacum*, gaps could result from the limitations of the assembly software as described above, or from structure variation such as large InDels between the target and the reference.

In terms of the errors observed in assemblies guided by distant references such as *A. thaliana* and *N. tabacum*, the error rates were substantially lower than those of 0.056% reported by Cronn et al. (2008) and 0.037% reported by Moore et al. (2006). The fact that no errors were observed for Chiifu-402-41 could be because of the very evenly distributed sequencing depth for this accession.

We sequenced whole cellular DNA using one accession per Solexa lane, which resulted in sequence redundancy reaching an average read density of 103× to 550× for each base of the cp genome. These sequencing depths could be over-estimated due to reads from homologous fragments in the chromosomal genome or mitochondrial genome, and also due to reads from repeated regions of cp genome. However, the true sequencing depth is more than sufficient for cp genome assembly. A previous study showed that sequencing depths greater than 10-fold had little effect on genome coverage (Li et al., 2008). This indicates that with the present sequencing strategy, a reasonable sequencing depth and coverage can be achieved by multiplex sequencing in one lane. This will considerably reduce sequencing costs, especially considering that new developed Solexa sequencing techniques can generate approximately 35 Gb of 150-bp paired-end reads from one lane.

In addition to assembly of the cp genome, the micro-reads produced using this approach could be used to identify SNPs in species for which the whole genome sequence is available, because most of these reads are from the chromosomal DNA sequence. This could be an additional value for the data obtained using this approach.

## Conflict of Interest Statement

The authors declare that the research was conducted in the absence of any commercial or financial relationships that could be construed as a potential conflict of interest.

## Supplementary Martial

The Supplementary Material for this article can be found online at http://www.frontiersin.org/Plant_Genetics_and_Genomics/10.3389/fpls.2012.00243/abstract

Supplementary Table S1 **Gaps in assemblies guided by cp sequences of *B. rapa*, *A. thaliana*, or *N. tabacum***.

Supplementary Table S2 **Annotated genes in *B. rapa* cp genome**.

Supplementary Table S3 **Errors and insertion/deletions (InDels) in assemblies guided by cp sequence of *A. thaliana* or *N. tabacum***.

Supplementary Table S4 **Single nucleotide polymorphisms (SNPs) and insertion/deletions (InDels) identified for *B. rapa* accessions Chiifu-402-41, Z16, and FT**.
